# Novel PEG-Modified Hybrid PLGA-Vegetable Oils Nanostructured Carriers for Improving Performances of Indomethacin Delivery

**DOI:** 10.3390/polym10060579

**Published:** 2018-05-24

**Authors:** Jana Ghitman, Raluca Stan, Adi Ghebaur, Sergiu Cecoltan, Eugeniu Vasile, Horia Iovu

**Affiliations:** 1 Advanced Polymer Materials Group, University Politehnica of Bucharest, 1-7 Gh Polizu Street, 011061 Bucharest, Romania; ghitmanjanusik@yahoo.com (J.G.); ghebauradi@yahoo.com (A.G.); sergiu.cecoltan@gmail.com (S.C.); 2 Faculty of Applied Chemistry and Material Science, University Politehnica of Bucharest, 1-5 Gh. Polizu Street, 011061 Bucharest, Romania; rl_stan@chim.upb.ro; 3 Department of Oxide Materials Science and Engineering, University Politehnica of Bucharest, 1-7 Gh. Polizu, 060042 Bucharest, Romania; eugeniuvasile@yahoo.com; 4 Academy of Romanian Scientists, 54 Splaiul Independentei Street, 050094 Bucharest, Romania

**Keywords:** hybrid nanocarriers, vegetable oils, indomethacin, PEG surface modification, in vitro release kinetics

## Abstract

The purpose of this work was to more exhaustively study the influence of nanocarrier matrix composition and also the polyethylene glycol (PEG)-modified surface on the performances of formulations as lipophilic drug delivery systems. Poly (d,l-lactide-*co*-glycolide), two vegetable oils (Nigella sativa oil and Echium oil) and indomethacin were employed to prepare novel PEG-coated nanocarriers through emulsion solvent evaporation method. The surface modification was achieved by physical PEG adsorption (in the post-production step). Transmission electron microscopy (TEM) nanographs highlighted the core-shell structure of hybrid formulations while scanning electron microscopy (SEM) images showed no obvious morphological changes after PEG adsorption. Drug loading (DL) and entrapment efficiency (EE) varied from 4.6% to 16.4% and 28.7% to 61.4%, solely depending on the type of polymeric matrix. The oil dispersion within hybrid matrix determined a more amorphous structure, as was emphasized by differential scanning calorimetry (DSC) investigations. The release studies highlighted the oil effect upon the ability of nanocarrier to discharge in a more sustained manner the encapsulated drug. Among the kinetic models employed, the Weibull and Korsmeyer-Peppas models showed the better fit (*R*^2^ = 0.999 and 0.981) with *n* < 0.43 indicating a Fickian type release pattern. According to cytotoxic assessment the PEG presence on the surface increased the cellular viability with ~1.5 times as compared to uncoated formulations.

## 1. Introduction

The use of a suitable drug delivery system has become an important approach for emerging valuable therapy. Versatile strategies are being followed to enhance the functionality and in vitro or in vivo performances of drug delivery devices.

The hybridization process of poly (d,l-lactide-*co*-glycolide) (PLGA) nanoparticles is one of the most promising tools for improving their efficacy in drug delivery applications [1]. The concept of hybrid formulations which result by physical association of PLGA nanoparticles and different vegetable/synthetic oils is a new trend in obtaining versatile carriers. Kumar et al. developed a hybrid nanosystem with core-shell architecture by combining the mechanical features of PLGA and high drug loading property of vegetable oil into a single formulation. The hybrid systems showed a notable loading capacity of (29.2% *w/w*) and also an improvement in control release capability, antioxidant activity and stability as compared to standard PLGA nanoparticles [2]. In two other papers, M. Narvekar et al. described the successful incorporation of lipophilic ATRA (all-*trans*-retinoic acid) into PLGA-synthetic oil nanoparticles (PONC). The authors reported not only a higher encapsulation efficiency of ATRA into hybrid nanocarriers but also more sustained release profile, avoiding the risk of uncontrolled initial burst release. These performances were attributed to the oil inclusion which introduced nanostructure into the polymeric matrix of the carrier. The extensive apoptotic cell death and the substantial anti-tumorigenic effects of ATRA-PONC (IC_50_ of ATRA-PONC: 2 μg/mL versus free ATRA: 17.5 μg/mL) in ovarian cancer cell subline SKOV-3PR were owed to higher cell permeation by the well-dispersed drug/oil gradually released from PONC [3,4].

Additionally, besides safety and effectiveness, the most important particularity of the vegetable oils-mediated synthesis of hybrid nanocarriers is the endowment with different biological activities such as antibacterial, anti-inflammatory, or antioxidant. Lacatusu et al. studied the effectiveness of lipid nanoparticles based on different natural oils (grape seed oil, fish oil, and laurel leaf oil) in counteracting free radicals. The oxidative damage of new bioactive nanocarriers was assessed against two tumor cells (MDA-MB 231, HeLa cell lines), and two normal cells (L929, B16 cell lines) measuring the total antioxidant activity. The results highlighted a scavenging capacity about 98% of oxygen free radicals for the formulations obtained by association of grape seed and laurel leaf oils and also a drastic decrease in tumor cell proliferation [5]. Moreover, Nigella sativa oil, a well-known vegetable oil with multiple therapeutic properties and plasmid DNA were co-encapsulated into chitosan modified-PLGA nanoparticles for gene therapy in Alzheimer’s disease. The authors suggested that the oil presence within nanocarriers could enhance treatment benefits for Alzheimer’s disease by promoting the neurite extension, a crucial process for neuroregeneration [6].

Similar to standard PLGA nanoparticles, the surface of hybrid formulations can be modified (PEGylated) to optimize their physical characteristics.

Polyethylene glycol (PEG) is a hydrophilic, nonionic, biocompatible polymer. The studies in the field show that the surface modification of preformed nanoparticles with PEG increases their aqueous solubility and stability, reduces the cytotoxicity, and prolongs their systemic circulation time [7,8]. PEG can be added to the surface of nanoparticles through different routes: covalent binding [9], mixing during the synthesis process [10], or surface adsorption in the post-production step [11].

Nigella sativa oil (NSO) is one of the most extensively used vegetable oil in the medical field owing to its remarkable therapeutic properties such as: anti-inflammatory [12], antibacterial, and anticancer [13].

Echium oil (EQ) is a vegetable oil extracted from the *Echium plantagineum* seeds, with significant amounts of omega-3, omega-6 fatty acids, stearidonic acid, and γ-linolenic acid [14]. The omega-3, omega-6 long chain polyunsaturated fatty acids are widely recognized as playing a role in regulating many inflammatory disorders like atherosclerosis, cancer, and rheumatoid arthritis [15].

Indomethacin (IMC) is a non-steroidal anti-inflammatory agent with analgesic and anti-pyretic activities [16] frequently used for the treatment of autoimmune and chronic inflammatory diseases [17]. Some epidemiological studies have indicated that IMC plays a role in reducing the risk of developing cancer [17,18]. Nevertheless, IMC appears to have high incidence of gastrointestinal side effects such as ulceration and hemorrhage [16]. Likewise, IMC is generally a highly crystalline drug with low solubility in water [19]. The micro-/nanoencapsulation of IMC within different polymeric carriers for improving its solubility and reducing side effects has been emphasized in many papers.

Aiming to obtain an amorphous dispersion of IMC molecules within carrier matrix, Kang et al. prepared IMC-loaded PLLA/PLGA microparticles, using supercritical fluids technique as an alternative IMC delivery system. The in vitro release studies highlighted a higher rate of IMC dissolution and also a noteworthy increase in cellular biocompatibility of IMC-loaded into formulations as compared to free IMC was revealed by in vitro cytotoxicity assays [16]. Dupeyron et al. used a multilevel factorial design to investigate the effect of polymeric matrix and surfactant (PEG, polysorbate 80) in the synthesis of nanocarriers with increased loading capacity and bioavailability of IMC. The authors reported an encapsulation efficiency higher than 50%. Mainly, the amount of PEG used in the formulation process showed to be the parameter with the greatest influence on the encapsulation efficiency (95%) owing to its co-surfactant behavior [20]. Moreover, Tomoda et al. emphasized the superiority of IMC-loaded PLGA nanoparticles obtained by antisolvent diffusion method with preferential solvation (without stabilizing agent) instead of those obtained by standard emulsion evaporation method in the presence of PVA-surfactant for iontophoretic transdermal delivery. The new formulations presented a ~59–68 times higher stability and electrophoretic mobility compared with PVA-coated PLGA nanoparticles, while ex vivo skin permeability testing highlighted the high potential of formulations without surfactant to be affected by electrical energy (iontophoresis) [21].

The objective of the current research study was to prepare new PEG-coated hybrid formulations from biodegradable PLGA and two vegetable oils (NSO and EQ) as novel IMC delivery systems. The formulations were prepared by solvent evaporation method and surface modification was achieved by non-covalent PEG adsorption (in the post-production step). In this way, the main properties of each employed component were cumulated into one hybrid entity to design a device with optimal features for improving stability and, at the same time, reducing side effects of encapsulated lipophilic drug (Indomethacin).

The performances of new hybrid nanocarriers were evaluated by studying more profoundly the impact of the vegetable oil’s type upon the structural integrity of polymeric matrix and also the effect of PEGylation process. The oil dispersion within the polymeric matrix forms a less ordered hybrid nanostructure allowing a higher drug loading capacity and more controlled release as compared to standard nanoparticles, while the high biocompatibility of PEG layer absorbed on the surface of the formulations reduces their side effects. The physical characteristics of the obtained hybrid systems were investigated through different techniques, like dynamic light scattering (DLS), SEM and TEM, and DSC. Infrared (FTIR) and ultraviolet (UV)-visible (Vis) spectroscopy were employed for physical characterization and release studies; also the cytotoxicity assessment of the nanocarriers was performed using the tetrazolium-based colorimetric assay (3-(4,5-dimethylthiazol-2-yl)-2,5-diphenyltetrazolium bromide)-MTT test.

## 2. Material and Methods

### 2.1. Materials

Poly (lactic-*co*-glycolic) acid 50:50, acid terminated, with mol wt 30,000–60,000 Da (PLGA) polyvinyl alcohol with mol wt 30,000–70,000 Da, 87–90% hydrolyzed (PVA), polyethylene glycol with mol wt 3000 Da (PEG), dichloromethane (DCM) > 99.9%, indomethacine (IMC) > 99.9%, trypsin EDTA 1X, penicillin-streptomycin 1X, dialysis sacks with avg. flat width 35 mm, and MWCO 12.000 Da were provided by Sigma-Aldrich, Taufkirchen Germany. Nigella sativa oil (NSO) was extracted in our laboratory from Nigella seeds (Junagadh, India) and Echium oil (EQ) was supplied by SC Herbavit SRL, Bucharest, Romania. The fatty acids content of the oils has been determined by gas chromatography (gas-chromatograph Agilent Technologies, 7890 A, Wilmington, DE, USA) and the main components are presented in Table 1. Phosphate buffer saline, PBS solution pH = 7.4 (8.0 g NaCl, 0.2 g KCl, 1.44 g Na_2_HPO_4_, 0.24 g KH_2_PO_4_) prepared in our laboratory, Bucharest, Romania. All chemicals were used without further purification.

### 2.2. Methods

#### The Preparation of IMC-Loaded Hybrid Polymeric-Oil Nanoparticles and Standard Nanoparticles

All formulations were obtained by standard emulsion solvent evaporation method [22,23]. A specific quantity of indomethacin, polymer and oil (NSO or EQ) was dissolved in 4 mL DCM and the obtained solution was homogenized through sonication (Vibra-Cell CVX 130, 20 kHz, 220 V, Newtown, CT, USA) for 5 min in an ice bath. Next, the organic phase was slowly added to 20 mL aqueous solution of 0.5% *w/v* PVA under constant stirring. The mixture was subjected to sonication for 15 min; afterwards it was stirred 3 h to evaporate the organic solvent. The obtained formulations were separated from the aqueous phase by centrifugation. The unbound PVA, unencapsulated oil, and drug were removed from the system by washing three times with ultra-pure water. Standard nanoparticles loaded with IMC (PLGA-IMC) were obtained following the same procedure except that the oil component was not included (Table 1).

### 2.3. Nanoparticles Surface Modification (Pegylation)

**Figure 1:**
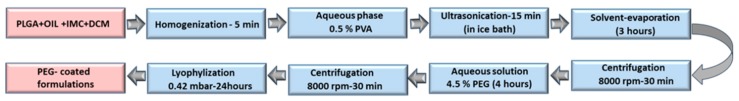
The chart flow of the preparation process.

All IMC-loaded standard or hybrid nanoparticles were incubated in a 4.5% *w/v* PEG3000 solution for 4 h under constant stirring, the formulations were then washed to remove unbound PEG (Figure 1) and were used freshly or lyophilized (Freeze Dryers, D-37520, Osterode am Harz, Germany) for further experiments.

### 2.4. Characterization

#### 2.4.1. Size Distribution, Zeta Potential, and Morphology

The particle size, polydispersity index (PdI), and zeta potential (ZP) of the nanoparticles were determined by dynamic light scattering using a Malvern Zeta Sizer ZEN 3600 (Worcestershire, UK, DLS). The samples were diluted in distilled water and 12 successive cycles were run at 25 °C, all the measurements were done in triplicate.

The morphology of the nanosystems was investigated by TEM and SEM. For TEM investigations, a small amount of each sample was deposited on a TEM copper grid covered with a thin amorphous carbon film with holes. After that, the TEM grid was placed on a sample holder and the geometrical evaluation (size and shape) of the samples was investigated by high-resolution transmission electron microscopy (HR-TEM) using a TECNAI F30 G2 S-TWIN instrument (Hillsboro, OR, USA).

SEM analyses were performed using a Quanta Inspect F50 (Hillsboro, OR, USA), with a field emission gun (FEG) with 1.2 nm resolution. The dried samples were sputter-coated with a thin gold layer and their morphology was observed with a field emission gun operated at 30 kV.

#### 2.4.2. Drug Loading (DL) and Encapsulation Efficiency (EE)

The freshly prepared formulations (PLGA-IMC, HPON-IMC, and HPOE-IMC) were washed three times with ultra-pure water to remove the unencapsulated drug, oils, and unbound PVA. After removing the supernatant, nanoparticles were dissolved in 10 mL DMSO and the amount of drug was determined spectrophotometrically using UV-Vis NIR spectrometer (Shimadzu, Kyoto, Japan) (UV-3600) at 318 nm for IMC (*y* = 17.587*x*, *R*^2^ = 0.9999). The experiments were repeated in triplicate, and the DL and EE (%) were calculated according to the Equations (1) and (2) respectively [2,16].

(1) $$\mathrm{DL}\;\left(\%\right)=\frac{Amount\;of\;drug\;entrapped\;}{Amount\;of\;NP}\times 100\%$$

(2) $$\mathrm{EE}\;\left(\%\right)=\frac{Amount\;of\;drug\;entrapped}{Amount\;of\;drug\;added}\times 100\%$$

#### 2.4.3. DSC Analyses

The changes in the crystalline state of formulations were studied by differential scanning calorimetry (DSC). Analyses were carried out using a Netzsch DSC 204 F1 Phoenix (NETZSCH-Gerätebau GmbH, Selb, Germany) differential scanning calorimeter under a constant nitrogen flow rate (20 mL/min) at a heating rate of 5 °C/min from 20 to 200 °C. Samples each containing 4–4.5 mg lyophilized nanoparticles, were placed in the aluminum pans, hermetically sealed, and equilibrated at 20 °C for 5 min. From collected DSC thermograms, the physical state of both nanocarrier matrix and entrapped drug were analyzed.

#### 2.4.4. FTIR Measurements

FTIR measurements were performed on a Vertex 70 Bruker FTIR spectrometer (Billerica, MA, USA) equipped with an attenuated total reflectance (ATR) accessory. For all uncoated or coated formulations the FTIR spectra were registered in the ATR-FTIR mode, at a resolution of 4 cm^−1^ in 600–4000 cm^−1^ wavenumber region. The measurements were done in triplicate.

#### 2.4.5. Release Studies

The in vitro release studies of IMC from PLGA-IMC, HPON-IMC, and HPOE-IMC, as well as PLGA-IMC-PEG, HPON-IMC-PEG, and HPOE-IMC-PEG were performed in phosphate buffer saline (PBS) at pH = 7.4 [24]. Thereby, a quantity of lyophilized samples corresponding each to 350 µg of drug was resuspended in 1 mL PBS and placed into pretreated dialysis cellulose bags (MWO = 1200 Da). Sealed bags containing samples were immersed into 20 mL of PBS under constant magnetic stirring (100 rpm) at 37 °C and, at predetermined time intervals, 3 mL volumes of samples were withdrawn and spectrophotometrically analyzed at 318 nm. In order to maintain the sink conditions, a volume of 3 mL of fresh PBS was periodically added. The concentration of released IMC was calculated by plotting the calibration curve of the drug in the range of 0.001–0.05 mg/mL (*y* = 20.993 + 0.0327*x*, *R*^2^ = 0.9994). The experiments were repeated in triplicate

#### 2.4.6. In Vitro Drug Release Kinetics and Mechanism

The in vitro IMC release data from all studied nanocarriers were evaluated kinetically using several standard mathematical models like first order, Higuchi, Hixson-Crowell, Korsmeyer-Peppas, and Weibull [25,26]

1. First order model

*Q* = *Q*_0_ · *e*^kt^

2. Higuchi model

*Q* = *k* · *t*^0.5^

3. Hixson-Crowell model

*Q*^1/3^ = *kt* + *Q*_0_^1/3^

4. Korsmeyer–Peppas model

*Q* = *k* · *t*^n^

5. Weibull model

*Q* = 1 − exp [−(*t*)^b/a^]

where *Q* is the amount of drug released in time *t*, *Q*_0_ is the start value of *Q*, *k* is the rate constant, *n* is the diffusional exponent (an indicative of drug release mechanism), *a* is the time constant, and *b* is the shape parameter.

The accurateness and prediction abilities of the employed models were compared by calculating the squared correlation coefficient (*R*^2^) and the Korsmeyer-Peppas model was employed to identify the balance between competing release mechanisms [27] (Table 2).

#### 2.4.7. Cytotoxicity Assay

The cytotoxic effect of IMC loaded into plain or hybrid uncoated or PEG-coated formulations was evaluated using (3-(4,5-dimethylthiazol-2-yl)-2,5-diphenyltetrazolium bromide) MTT metabolic assay on L929 cells line (Cell culture ECACC, fibroblasts from mouse adipose tissue) [16,28]. The cells were seeded in 96-well plates with a density of 1 × 10^5^/mL and incubated for 24 h at 37 °C in a humidified atmosphere of 95% air and 5% CO_2_ to allow cell attachment. Afterwards, the cells were incubated at 37 °C for 24 h with different IMC concentrations in the form of free drug or loaded into formulations followed by incubation with 5 mg/mL MTT solution for additional 3 h at 37 °C in the same atmosphere. Then, the medium was replaced with 100 µL acidified buffer (10% SDS, 0.01% HCl, 50% DMF) and incubated overnight at 37 °C under orbital shaking to solubilize the purple formazan crystals produced by the metabolically active cells. The quantification of cell viability was accomplished by measuring the absorbance at 570 nm with a microplate reader (Tecan M Infinit Nanoquant, Männedorf, Switzerland) using the untreated cells as positive control. The measurements were repeated four times.

The percent of cell viability was calculated using Equation (3)

(3) $$\mathrm{Cell}\;\mathrm{viability}\;\left(\%\right)=\frac{Absorbance\;intensity\;of\;the\;treated\;cells}{Absorbance\;intensity\;of\;the\;non-treated\;cells}\times 100\%$$

#### 2.4.8. Statistical Analyses

The data are expressed as mean ± SD. The significance of differences was evaluated by single factor ANOVA test and it was considered significant if *p* < 0.05.

## 3. Results and Discussion

### 3.1. Morphology, Hydrodynamic Characteristics, DL, and EE

Free or IMC-loaded nanocarriers were prepared using two types of vegetable oils one more saturated (NSO) and the other with a higher degree of unsaturation (EQ). The following IMC-loaded nanocarriers were prepared: PLGA-IMC (IMC encapsulated into PLGA standard nanoparticles without surface modification), HPON-IMC (IMC incorporated into hybrid nanoparticles containing 1/1 wt PLGA/NSO ratio), and HPOE-IMC (IMC loaded into hybrid nanoparticles containing 1/0.5 wt PLGA/EQ ratio); as well as PLGA-IMC-PEG, HPON-IMC-PEG, and HPOE-IMC-PEG (IMC-loaded into standard or hybrid systems coated with PEG). The hydrodynamic characteristics of obtained formulations are presented in Table 3.

Standard nanoparticles (PLGA-NP) with a mean size around 100 nm and moderate stability were obtained. The medium zeta negative potential was attributed to the end carboxylic groups from the surfaces of the polymeric matrix [29].

It was noted that the nature of encapsulated vegetable oil, the quantity of polyunsaturated fatty acids (EQ includes high amounts of ω-6 (C18:3, C18:4) and ω-3 (C18:2); NSO is rich in ω-6 (C18:2)) from oil structure, also the position and number of unsaturated double bonds exhibit a great influence upon the final features of hybrid nanoparticles. Thus, the addition of 1/1 wt NSO with respect to the polymeric matrix increased the dimension of HPON-NP with ~35% while the dispersion of only 1/0.5 wt PLGA/EQ ratio led to hybrid nanoparticles with ~45% higher size as compared to standard ones. The higher size of HPOE-NP versus HPON-NP could be correlated to different intern assembles of the long polyunsaturated fatty chains [5] which prevail in EQ and are in minority in NSO. Hybrid formulations presented a 30% lower size distribution and a significantly 80% higher stability than standard nanoparticles. This effect was attributed to the oil addition, which acts as a HLB moderator in the primary emulsification process, by increasing the HLB requirement of the system and forming a more stable and uniform emulsion.

The IMC loading tended to increase the size of nanocarriers. This trend was more obvious for PLGA-IMC (~55%) and less significant for hybrid formulations, for which the diameter increased with ~10% after IMC encapsulation. The low influence of IMC upon the mean size of hybrid polymeric matrix may be explained by the drug accommodation into more amorphous hybrid nanostructure as it was revealed by subsequent DSC tests.

It was interesting to note that the drug encapsulation meaningfully decreased the size distribution and stability of all nanosystems. Probably the addition of a supplementary lipophilic constituent (IMC) plays a role in the primary emulsification process determining the formation of a more uniform emulsion. The influence of lipophilic compound on the emulsification process of o/w emulsion was also reported by M.Y. Hasan et al. who investigated this phenomenon by constructing the ternary phase equilibrium diagram [30]. On the other hand, the decrease in stability may be explained by non-covalent interactions (hydrogen bonding) which appeared between IMC and PVA [31], and also between IMC and polymeric matrix (PLGA or vegetable oils). The PVA adsorption on the surface of formulations has been reported for other IMC loaded PLGA nanoparticles for which a dramatic decrease in stability was noted [32]. In this study, the reported data stressed the importance of the vegetable oil which not only increases the encapsulation capacity but also minimizes the negative influence of IMC upon the nanocarriers’ stability.

To confirm the DLS results and to study more thoroughly their morphology, the nanocarriers were assessed through TEM and SEM microscopy. Generally, both analyses highlighted spherical structures with smooth surface ranging ~150 nm.

**Figure 2:**
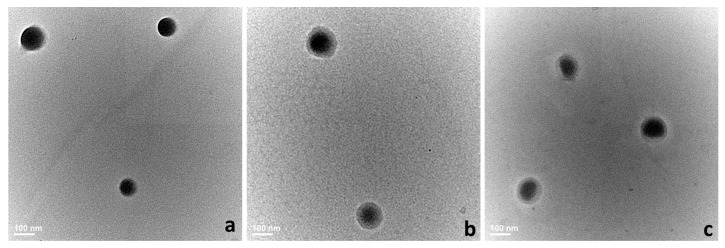
The morphology of analyzed systems evaluated through TEM microscopy. (**a**) PLGA-IMC; (**b**) HPON-IMC; and (**c**) HPOE-IMC.

TEM nanographs (Figure 2a–c) registered obvious differences in the surface morphology of standard and hybrid systems. Standard PLGA-IMC presented uniform, smooth and spherical structure (Figure 2a) while hybrid systems (Figure 2b,c) showed a well-defined core-shell structure which was depicted in TEM images as two different compartments: the oil core (dark color) surrounded by an uniform polymeric shell (bright color).

**Figure 3:**
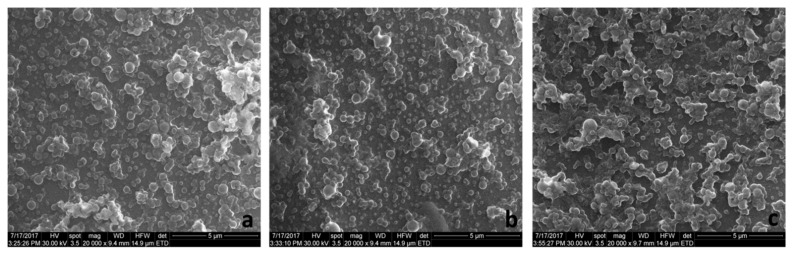
The SEM images of (**a**) PLGA-IMC; (**b**) HPOE-IMC; and (**c**) HPOE-IMC-PEG.

The SEM images of selected PLGA-IMC and HPOE-IMC are depicted in Figure 3a,b. The photographs revealed the formation of homogenous slightly agglomerate population with well-defined structure. The agglomeration tendency observed for all uncoated formulations could be explained by non-covalent IMC-PVA interactions which determine a decrease in stability of the systems, as previously reported in the literature [32] and was confirmed in our research study by DLS investigations.

The hydrophilic coating (PEG) led to a noticeable deterioration in the size (~67%) and polydispersity (~77%) of standard PLGA-IMC and did not affect significantly the mean size of hybrid devices (Table 3). Generally, the increasing size and distribution of the nanocarriers after PEG adsorption in a post-production step was attributed to the deposition of multilayer surface-coating component [11]. Therefore these parameters could be affected by the actual amount of unevenly deposited PEG on the surface of formulations which depend on the interactions between PVA, surfactant-entrapped IMC, and PEG. Perhaps, due to the low PLGA matrix-lipophilic drug affinity, the most of IMC was entrapped in the surface layers of the nanoparticles. As a result of interactions (such as hydrogen bonding) which appeared between the indole amide of IMC and PVA, [31] the coating process of PLGA-IMC-PEG was considerably affected.

The adsorption of PEG increased the PdI value of HPON-IMC from 0.109 ± 0.020, to 0.179 ± 0.004 and did not affect the PdI of HPOE-IMC (0.107 ± 0.020 to 0.108 ± 0.015). This fact could be correlated with encapsulation efficiency of IMC within hybrid formulations, which was almost double for HPON-IMC as compared to HPOE-IMC. Probably due to an increased amount of surfactant-entrapped drug encapsulated near to the surface, the hydrophilic interactions of hybrid matrix with PEG were affected and PdI thus increased.

In terms of stability, the surface modification with PEG shifted the ZP values towards less negative values (Table 3). As a consequence of PEG chains hydration, the diffuse layer was shifted to a larger distance from the particle and also multiple hydrogen bonds occurred determining a reduction of ZP values [20,33]. This fact suggests the probability of appearance of some agglomeration between the colloids. The registered SEM images of the coated systems supported this phenomenon, highlighting moderate agglomerated formulations with rather spherical surface and the mean diameter ~170 nm (Figure 3c).

In Table 4, the DL and EE values calculated for plain uncoated or hybrid nanoparticles are summarized. The lowest DL and EE values were noticed for standard nanoparticles as compared to hybrid formulations for which both values were approximately double (HPOE-IMC) or three times higher (HPON-IMC). The DL and EE can be influenced not only by the encapsulation method but also by the polymeric matrix-drug affinity. The increased values of DL and EE achieved for hybrid formulations could be due to the higher compatibility of lipophilic IMC with nanostructured hybrid lipophilic matrix [11,20] and also to the oil’s capability to stabilize the encapsulated lipophilic drug.

DL and EE for the PEG-coated formulations were considered the same. The surface modification of the nanocarriers was performed after drug encapsulation (in the post-production step) and, owing to the lipophilic character of IMC the drug leakages during the coating process, are negligible.

### 3.2. DSC Analyses

**Figure 4:**
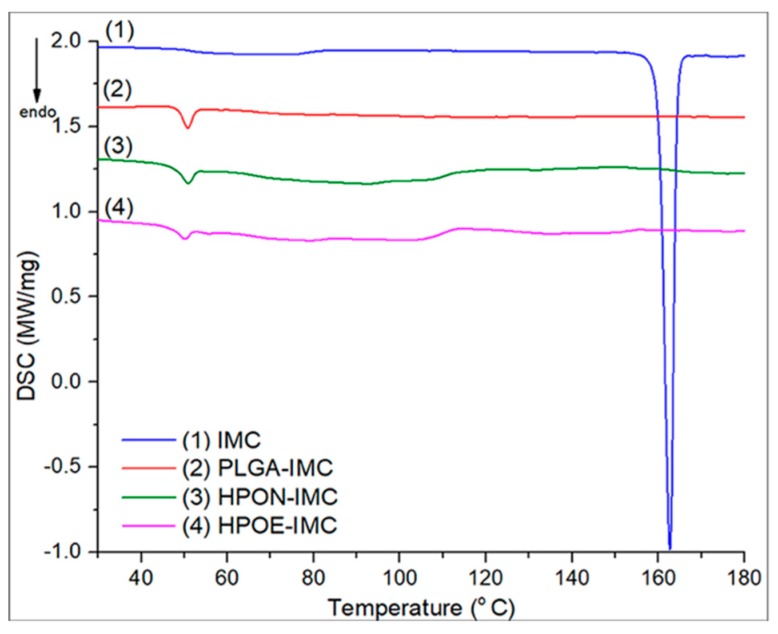
DSC thermograms of analyzed samples.

The assessment of the oil’s effect upon the polymeric matrix and IMC encapsulation into the nanocarriers was done by DSC analyses (Figure 4).

The DSC thermogram of PLGA-IMC revealed an endothermic peak at ~49.9 °C which was attributed to the polymeric matrix relaxation peak followed by the glass transition phase. Even if the selected oils have different composition in the polyunsaturated acids (EQ rich in ω-3 and ω-6, NSO in ω-6) the type and amount of dispersed oil within the polymeric matrix did not show a significant influence on the glass transition domain of hybrid formulations (for HPON-IMC *T*g value was ~49.9 and ~49.1 °C for HPOE-IMC, respectively).

Nonetheless, the areas of endothermic peaks which are proportional to enthalpy (∆*H*) changes showed that the oil incorporation into the polymer led to an increase in amorphosity (decrease its crystallinity). Highly disordered hybrid matrices were formed requiring a lower energy input to disrupt the more amorphous structures. ∆*H* of standard PLGA-IMC was 3.48 J/g, the incorporation of 1/1 wt NSO with respect to polymer matrix reduced it to 2.97 J/g while the incorporation of only 1/0.5 wt EQ decreased the enthalpy to 1.70 J/g. This effect was attributed to a high content of polyunsaturated fatty acids from EQ composition implying a larger space to be accommodated into the polymeric matrix [34].

The DSC thermogram of IMC exhibited a sharp endothermic peak at 162.7 °C corresponding to drug melting point [35]. After nanoformulation, the characteristic peak of IMC disappeared suggesting that the drug molecules were in a highly dispersed state in the nanocarrier matrix [36].

### 3.3. FTIR Studies

**Figure 5:**
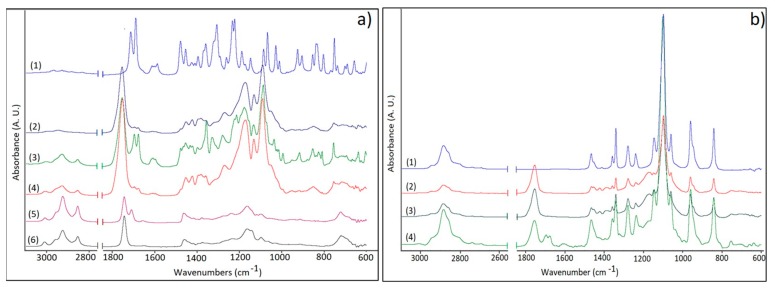
(**a**) FTIR spectra of (1)–IMC, (2)–PLGA-IMC, (3)–HPON-IMC, (4)–HPOE-IMC, (5)–NSO, and (6)–EQ; (**b**) FTIR spectra of PEG-coated formulations: (1)–PEG; (2)–PLGA-IMC-PEG; (3)–HPOE-IMC-PEG, and (4)–HPON-IMC-PEG.

The encapsulation of the drug and oils into plain or hybrid formulations and the PEG coating process were further confirmed through FTIR analyses (Figure 5a,b).

The spectrum of indomethacin showed characteristic bands of –C–H deformation vibration at 752 cm^−1^ from the aromatic rings, C–Cl stretching vibrations at 1068 cm^−1^, C–O stretching vibrations from phenyl groups at 1307 cm^−1^, C=C stretching vibrations at 1589 cm^−1^ from the aromatic rings, C=O (amide I) stretching vibrations at 1692 cm^−1^ and C=O secondary carbonyl groups at 1718 cm^−1^ from COOH (Figure 5a) [20,37].

The recorded spectra of the oils (NSO and EQ) highlighted representative stretching vibrations of *trans* olefinic double bonds of unsaturated acids at approximately 3006 cm^−1^ and the characteristic C=O stretching vibration bands of triglycerides at 1747 cm^−1^ [38]. The second sharp peak at 1711 cm^−1^ from NSO spectrum was attributed to carbonyl stretching vibrations from conjugated ketone structures, the therapeutic compounds from oil composition (thymoquinone, ditymoquinone). As depicted in Figure 5a the HPON-IMC and HPOE-IMC spectra show the representative absorption bands at 3006 cm^−1^ from oils composition.

The characteristic absorption band of amide I and secondary carbonyl groups respectively, were shifted to lower values with ~20 cm^−1^ in all nanosystems. This effect can be a result of non-covalent interactions such as hydrogen bonding which appeared between the functional group of IMC structure (–COOH and –NC=O) and PVA [31] or between IMC and carboxyl (–COOH) groups from PLGA and oils structures. The distinctive C–Cl stretching vibration at 1068 cm^−1^ was registered as a small shoulder, being overlapped by the C–O bands at 1097 cm^−1^ of the esters groups from oils or PLGA structure.

The PEG presence on the surface of the nanocarriers was confirmed by identifying the characteristic absorption bands at 2884 and 1102 cm^−1^ representative for C–H stretching vibrations, as well as for C–O–C stretching vibrations of the repeated –OCH_2_CH_2_–units of the PEG structure [11,39] (Figure 5b).

### 3.4. In Vitro Drug Release Studies

**Figure 6:**
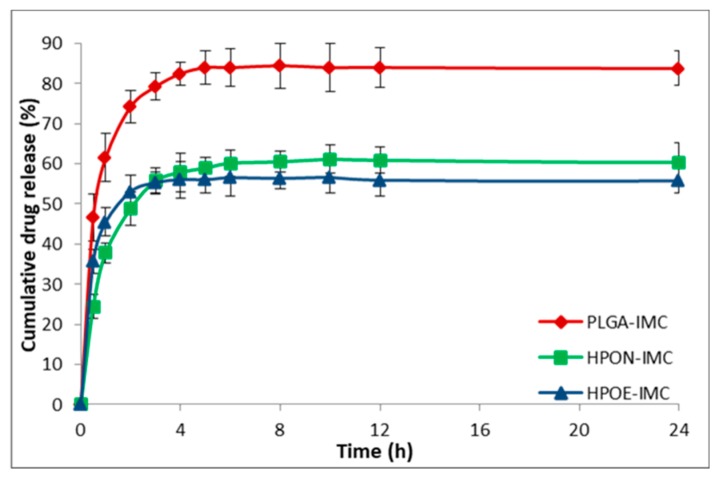
In vitro release profile of IMC from plain or hybrid uncoated formulations.

**Figure 7:**
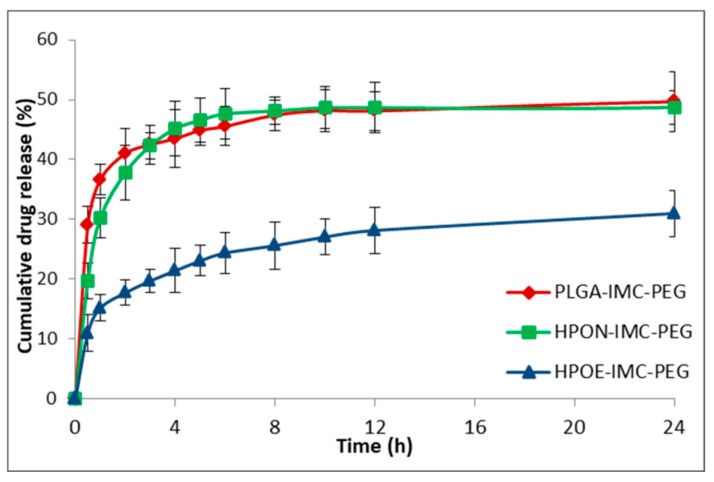
In vitro release profile of IMC from PEG-coated nanocarriers.

The in vitro release profile of IMC from all analyzed nanosystems is shown in Figure 6 and Figure 7. PLGA-IMC nanoparticles showed a standard diphasic release profile [23] characterized by initial burst release up to 46.7% in first 30 min (Figure 6) followed by a slower release phase (83.0%) after 24 h and remain constant for the next 72 h (data are not shown).

By introducing nanostructured oils into the polymeric matrix the initial burst release (HPON-IMC and HPOE-IMC) was visible suppressed (in the first 30 min the concentration of IMC released from HPON-IMC was ~24.4% and ~35.7% respectively from HPOE-IMC formulations) (Figure 6). A role in the initial burst release can be attributed to polymer hydrophilicity, a more hydrophilic polymeric matrix (PLGA-IMC) facilitated water uptake from the release medium, leading to a great burst effect and faster release as compared to a more lipophilic hybrid matrix [40,41].

Moreover, the initial burst release and the capacity of nanocarrier to discharge in a controlled manner the encapsulated drug solely depend on the polymeric matrix-drug affinity [42], which is higher between lipophilic IMC and hybrid lipophilic matrix than IMC and standard polymeric matrix. The progressively slow release rate of IMC from hybrid formulations (the concentration of IMC from release medium was ~60.3% in the first 24 h and increased to ~61.0% after 72 h in the case of HPON-IMC and ~55.8% after 24 h and ~58.0% after 72 h were registered for HPOE-IMC) suggested that the high amorphous hybrid structure (as highlighted DSC results, Figure 4) allowed a more uniform distribution of the entrapped drug molecules within the polymeric matrix which was stabilized into the oil core.

Concerning the coated formulations (Figure 7) the adsorption of PEG on the surface determined a decrease of IMC release rate compared to the corresponding uncoated systems.

The IMC release from surface modified PLGA-IMC-PEG, HPON-IMC-PEG, and HPOE-IMC-PEG formulations were ~49.7%, ~48.7%, and ~31.0% in the first 24 h achieving concentrations of ~58.8%, ~56.0%, and ~35.5% correspondingly in 72 h. This behavior could be attributed to the presence of PEG layer on the surface acting as an additional barrier to drug diffusion [41].

### 3.5. In Vitro Drug Release Kinetic Mechanisms

The drug release mechanism from nanocarrier matrix could be classified either in diffusional or erosion controlled, or can be governed by both mechanisms [43]. Therefore, to obtain a clear view of the IMC release, the data obtained from the dissolution profile were fitted to different mathematical models such as first order, Higuchi, Hixson-Crowell, Korsmeyer-Peppas, and Weibull.

**Figure 8:**
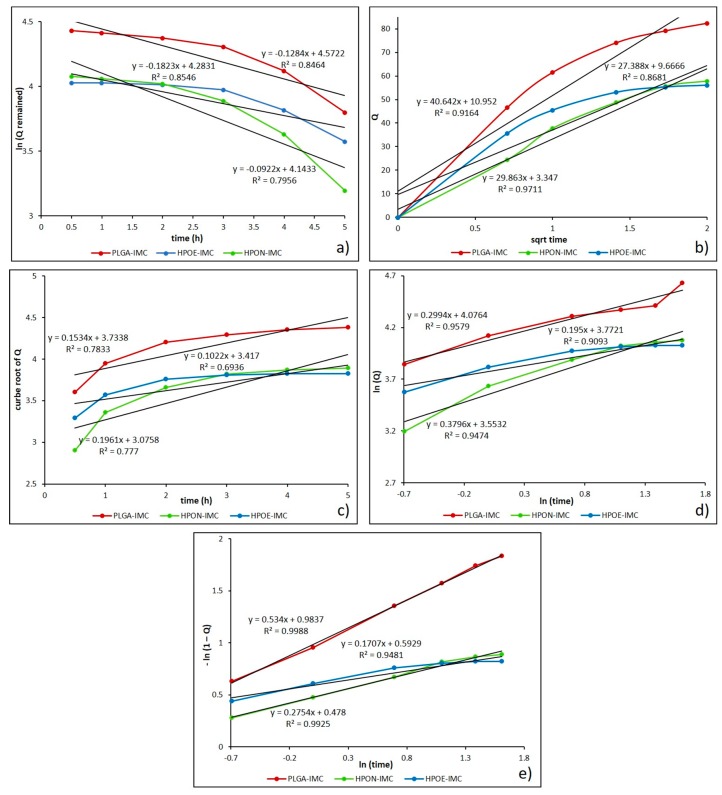
Drug release data fitted to various kinetic models. (**a**) First order; (**b**) Higuchi model; (**c**) Hixson-Crowell model; (**d**) Korsmeyer-Peppas model; and (**e**) Weibull drug release model.

In Table 5 are summarized the relevant parameters characterizing the release kinetic models and also in Figure 8 are depicted the IMC release data from standard (PLGA-IMC) and hybrid formulations (HPON-IMC and HPOE-IMC) fitted to mathematical models.

Initially, the obtained dissolution data were fitted into the first order kinetic equation (log cumulative percent of drug remaining versus time) and the regression parameters (*R*^2^) values were found in the range 0.749–0.931, suggesting that the release kinetic did not follow this mathematical model (Figure 8a). Generally, first order model is derived from first order release kinetics, describing the systems where dissolution rate is directly dependent on the concentration of the dissolving species [44].

Next, the mechanism of drug release from the nanocarrier matrix was evaluated by fitting the obtained dissolution data to Higuchi equation (cumulative percentage of drug release versus square root of time). As presented in Figure 8b and Table 5, the data were more appropriated to this model (*R*^2^ = 0.766–0.971), which indicate that the release of IMC from drug delivery systems seems to be a process predominately controlled by diffusion [44,45]. Likewise, to establish if the erosion process was also involved in the drug release kinetics, the data obtained from in vitro release studies were plotted as cube root of released drug versus time for Hixson-Crowell model (Figure 8c). The relatively low values calculated for the regression parameter (0.693 to 0.880) (Table 5) suggest that erosion process of polymeric matrix is not the main one, as in generally cases of PLGA biomaterials in simulated body fluids [46].

To further confirm that the drug release mechanism is a process predominately controlled by diffusion, the data were fitted into Korsmeyer-Peppas equation (log cumulative percentage drug release versus log time) (Figure 8d). From the results presented in Table 5, it can be observed that all the formulation showed a good correlation (*R*^2^ = 0.938–0.981) to this mathematical model. The diffusional exponent (*n*) and the rate constant (*k*) from Peppas model are the more important parameters which are overseen by the release mechanism. The value of diffusional exponent (*n*) is a parameter which specifies the drug release mechanism from polymeric dosage forms when more than one type of release phenomenon was involved. According to n value the drug release from a dosage form may occur through Fickian diffusion if *n* < 0.43, when 0.43 < *n* < 0.85 it indicates a non-Fickian type release (a combination of both diffusion and erosion mechanisms) also a greater value of *n* than 0.85 indicates the Case II transport [26,47,48]. The calculated *n* values were in the range from 0.178 to 0.380, suggesting a Fickian release (diffusion-controlled release) which occurred through the molecular diffusion of the drug based on the chemical potential gradient [47].

Moreover, high value of rate constant (*k*) can suggest a faster discharge of the drug from nanoparticles characterized by a great initial burst release [26,43]. From Table 5, it can be seen that *k* values of formulations decreased from 62.452 to 34.925 with the oil addition, and from 34.713 to 14.216 after PEG coating process respectively. The decrease in rate constant parameter denotes that the drug release kinetics became slower and more controlled, in concordance with release studies.

Finally, the best results were obtained when the release data were fitted into Weibull model (*R*^2^ = 0.948–0.999) which describes the dissolution profile in terms of shape (*b*) and scale parameters (Figure 8e). The *b* parameter is a characteristic of the shape of the dissolution curve, thus when *b* = 1 it indicates an exponential profile, *b* > 1 represents a sigmoidal curve with a turning point and *b* value lower than 1 indicates a steeper initial slope than is consistent with exponential profile [25]. Determined Weibull parameter (*b*) was lower than 1 for all analyzed systems, specifying a parabolic curve with a sharper initial slope than is similar with the exponential profile (Table 5).

In the literature there are many studies completed on dissolution comparison of low solutbility drugs or drug profiles from different types of carrier matrices, highlighting that the Weibull model is suitable for characterizing the dissolution kinetics [25,26,49].

### 3.6. Cytotoxicity Studies

**Figure 9:**
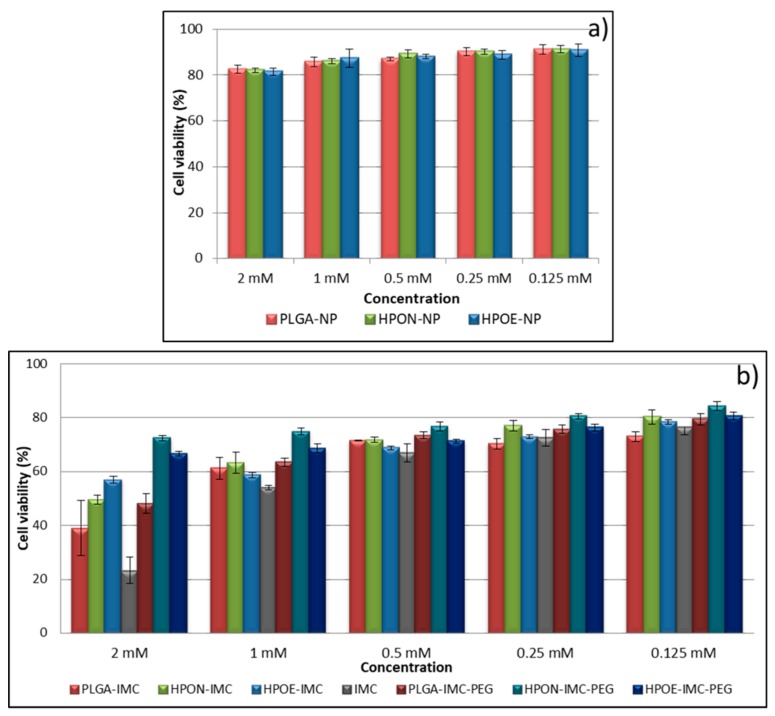
In vitro viability of L929 fibroblast cells line treated with various concentrations of (**a**) empty nanocarriers and (**b**) IMC- loaded into formulations.

The aim of MTT assessments were to determine the intrinsic cytotoxicity of IMC-loaded into plain or hybrid unmodified or PEG-modified formulations consecutively to obtain a preliminary estimation of the safety of new nanoformulations (Figure 9a,b).

It is known that PLGA is one of the most used polymers in drug delivery systems, approved by the FDA, due to its biocompatibility and biodegradability [1,16,23]. However, to exclude the possible cytotoxicity of prepared formulations, empty nanocarriers (PLGA-NP, HPON-NP, and HPOE-NP) were also evaluated in vitro by cytotoxicity assays (Figure 9a). The obtained results revealed that the cell viability and proliferation was not significantly affected by the tested nanoparticles. The cell viability values were found to be above 80% for all the tested samples, thus indicating the lack of cytotoxicity of empty carriers and the possibility to be used as vehicles for drugs.

Figure 9b depicted the cytotoxic response of L929 fibroblast cells line evaluated after 24 h in vitro at different IMC concentrations in the form of free drug or loaded into the formulations.

At starting concentration (2 mM), free IMC revealed an advanced cytotoxicity effect on fibroblast cells (~78%) which was diminished with sample dilution.

Concerning the IMC nanoformulation, plain PLGA-IMC showed advanced cellular growth inhibition compared to all hybrid formulation. The viability of fibroblasts exposed to 2 mM drug concentration in the form of PLGA-IMC was only ~39%, while an increase in cellular viability of the fibroblasts incubated with hybrid nanostructured polymer-oil matrices loaded with IMC was registered (two times higher for HPON-IMC and ~2.4 times for HPOE-IMC at the same drug concentration).

The high cell growth inhibition of the PLGA-IMC as compared to cytotoxic effect of hybrid systems is probably related to the low dispersion and stability of IMC molecules into the polymeric matrix. The oil dispersion within more amorphous hybrid matrices improved the distribution and stability of lipophilic drug, leading to a gradually controlled IMC release and a low toxicity.

Regarding the coated formulations, as it was expected that the PEG adsorption on the surface of the nanocarriers visibly increased the cellular biocompatibility, registering high rates of cells survival [7,50].

## 4. Conclusions

New hybrid nanocarriers from biodegradable PLGA and two vegetable oils (Nigella sativa oil and Echium oil) for improving stability and loading capacity of Indomethacin were prepared using solvent evaporation method. The surface modification of the nanocarriers was achieved by physical PEG adsorption (in the post-production step).

Hydrodynamic studies highlighted formulations with a mean diameter around 160–167 nm and narrow size distribution (PdI ≤ 0.13). TEM images showed clearly the core-sell structure of hybrid formulations, while SEM images depicted no obvious changes in the morphological features of plain and PEG-coated formulations. The amount of the PEG absorbed on the surface was influenced by the surfactant-IMC interactions, as well as by the type of the nanocarrier matrix.

Oil incorporation within the polymeric matrix led to a more amorphous nanostructure which enhanced the drug loading capacity of the devices, but the relation between the loading capacity of nanocarrier and the type of dispersed vegetable oil within polymeric matrix still remains unclear.

On the other hand, encapsulated oils influenced directly the ability of nanocarrier to discharge in a more sustained manner the entrapped drug by ensuring the optimum environment and stabilizing the lipophilic compound. A slower IMC release from coated formulations was attributed to the presence of PEG layer on the surface acting as an additional barrier to drug diffusion. Among the kinetic models employed, the Weibull and Korsmeyer-Peppas models showed the better fit (*R*^2^ = 0.999 and 0.981, respectively) with *n* < 0.43 specifying a release process predominantly controlled by diffusion (Fickian type release pattern). The *k* value (Peppas model) decreased with oil and PEG addition, confirming the reduction of the initial burst release. According to Weibull parameter (*b*) the shape of dissolution curve is parabolic with sharper initial slope than is similar with the exponential profile. The cytotoxic assessment highlighted that the oil dispersion within the polymeric matrix decreased the cell growth inhibition by ~2.4 times as compared to standard nanocarriers, while the PEG-adsorption on the surface of the formulations increased the cellular viability by ~1.5 times as compared to uncoated formulations.

## Figures and Tables

**Table 1:** Main composition of prepared standard or hybrid nanocarriers

Sample	Vegetable oils and its main components		Indomethacin IMC (wt %)
PLGA-NP	-	-	-
PLGA-IMC			10%
HPON-NP	Nigella sativa oil	62.84% linoleic acid (C18:2) 20.80% oleic acid (C18:1) 11.42% palmitic acid (C16:0) 1.61% stearic acid (C18:0) 1.23%cis-11,14-eicosad acid (C20:2) 0.16% myristic acid (C14:0) 0.10% palmitoleic acid (C16:1)	-
HPON-IMC (PLGA/NSO ratio 1/1 wt)			10%
HPOE-NP	Echium oil	33.79% α-linolenic acid (C18:3) 16.10% oleic acid (C18:1) 14.83% linoleic acid (C18:2) 14.32% stearidonic acid (C18:4) 10.74% γ-linolenic acid (C18:3) 6.93% palmitic acid (C16:0) 3.26% stearic acid (C18:0)	-
HPOE-IMC (PLGA/EQ ratio 1/0.5 wt)			10%

**Table 2:** Drug release mechanism according to n value from Korsmeyer-Peppas model

Release exponent (*n*)	Drug transport mechanism
*n* < 0.43	Fickian release (diffusion-controlled release)
0.43 < *n* < 0.85	Non-Fickian release (anomalous transport, combination of diffusion and erosion drug release mechanism)
0.85 < *n* < 1	Case II transport (relaxation-controlled release)
*n* > 1	Super case II transport (swelling and polymer chain relaxation controlled release)

**Table 3:** Hydrodynamic features of analyzed system

Formulation	*d* (nm)	PdI	ZP (mV)
PLGA-NP	106.0 ± 1.4	0.210 ± 0.016	−12.2 ± 1.2
HPON-NP	144.5 ± 0.7	0.163 ± 0.031	−22.0 ± 0.9
HPOE-NP	153.0 ± 2.5	0.164 ± 0.014	−22.1 ± 1.3
PLGA-IMC	165.2 ± 0.7	0.134 ± 0.008	−13.4 ± 0.1
HPON-IMC	160.9 ± 1.9	0.109 ± 0.020	−15.2 ± 0.4
HPOE-IMC	167.1 ± 1.8	0.107 ± 0.025	−14.4 ± 0.5
PLGA-IMC-PEG	275.8 ± 2.7	0.230 ± 0.003	−10.1 ± 0.2
HPON-IMC-PEG	179.0 ± 2.5	0.179 ± 0.004	−14.0 ± 0.8
HPOE-IMC-PEG	200.9 ± 2.2	0.108 ± 0.015	−13.6 ± 1.2

**Table 4:** DL and EE of the new prepared formulations

Formulation	DL (%)	EE (%)
PLGA-IMC	4.6 ± 0.2	28.7 ± 2.4
HPON-IMC	16.4 ± 0.4	61.4 ± 2.6
HPOE-IMC	8.5 ± 0.1	37.7 ± 1.9

**Table 5:** Kinetic modeling of IMC release from all analyzed formulations

Formulation	First order		Higuchi		Hixson-crowell		Korsmeyer-peppas			Weibull		
	*R*^2^	*k*	*R*^2^	*k*	*R*^2^	*k*	*R*^2^	*n*	*k*	*R*^2^	*a*	*b*
PLGA-IMC	0.846	0.147	0.916	47.490	0.783	0.153	0.957	0.213	62.452	0.998	0.983	0.534
HPON-IMC	0.749	0.136	0.971	30.212	0.777	0.196	0.943	0.380	34.925	0.992	1.613	0.275
HPOE-IMC	0.795	0.036	0.868	33.697	0.693	0.138	0.909	0.218	43.489	0.948	1.809	0.170
PLGA-IMC-PEG	0.854	0.066	0.766	26.266	0.733	0.073	0.979	0.178	34.713	0.971	1.548	0.101
HPON-IMC-PEG	0.854	0.129	0.950	24.690	0.780	0.169	0.976	0.259	30.898	0.994	1.419	0.170
HPOE-IMC-PEG	0.931	0.125	0.911	11.393	0.880	0.083	0.981	0.305	14.216	0.990	1.172	0.060
